# Plasmakinetic resection technology for the treatment of benign prostatic hyperplasia: evidence from a systematic review and meta-analysis

**DOI:** 10.1038/srep12002

**Published:** 2015-07-09

**Authors:** Sheng Li, Joey S.W. Kwong, Xian-Tao Zeng, Xiao-Lan Ruan, Tong-Zu Liu, Hong Weng, Yi Guo, Chang Xu, Jin-Zhu Yan, Xiang-Yu Meng, Xing-Huan Wang

**Affiliations:** 1 Department of Urology, Zhongnan Hospital, Wuhan University, Wuhan, People’s Republic of China; 2 Center for Evidence-based and Translational Medicine, Wuhan University, Wuhan, People’s Republic of China; 3 Chinese Evidence-Based Center and Chinese Cochrane Center, West China Hospital, Sichuan University, Chengdu, Sichuan Province, People’s Republic of China; 4 Department of Hematology, Renmin Hospital of Wuhan University, Wuhan, People’s Republic of China; 5 Department of Epidemiology, School of Public Health, Wuhan University, Wuhan, People’s Republic of China

## Abstract

The aim of this study was to compare plasmakinetic resection of the prostate (PKRP) with transurethral resection of the prostate (TURP) for benign prostatic hyperplasia (BPH) in terms of efficacy and safety. Published RCTs were searched from PubMed, Embase, Science Citation Index, and Cochrane Library up to April 10, 2014. After methodological quality assessment and data extraction, meta-analysis was performed using the STATA 12.0 software. 18 reports of 16 RCTs were included in this analysis. Meta-analyses showed that PKRP significantly improved Qmax at 12 months, but no significant difference was found for other efficacy outcomes. In terms of safety, treatment of PKRP was associated with reduced drop in serum sodium, lower TUR syndrome, reduced need of blood transfusion, clot retention, and shorter catheterization time and hospital stay; in contrast, there were no significant differences in the analysis of operative time, postoperative fever, and long-term postoperative complications. In summary, current evidence suggests that, although PKRP and TURP are both effective for BPH, PKRP is associated with additional potential benefits in efficacy and more favorable safety profile. It may be possible that PKRP may replace the TURP in the future and become a new standard surgical procedure.

Benign prostate hyperplasia (BPH) is the most common cause of urination obstacles in elderly men, and its incidence increases with the growth of age^1^. For many years, transurethral resection of the prostate (TURP) has been regarded as the gold standard for patients with lower urinary tract symptoms (LUTS) secondary to BPH who are in need of aggressive treatment or for whom medical therapy has failed^2^,^3^. However, the complications of bleeding and transurethral resection (TUR) syndrome associated with treatment of TURP often lead to death. In a recent study of 10,654 men who underwent TURP, peri-operative mortality (during the first 30 days) was 0.1%^4^. This prompted researchers to seek a safer method with less trauma. Bipolar transurethral resection technology (B-TURP) is one of the most important breakthroughs in the field of TURP. The 2013 European Association of Urology (EAU) guideline stated that the short-term profile of B-TURP was comparable to TURP. To date, there are five types of bipolar resection devices: the Plasmakinetic (PK) system (Gyrus), transurethral resection in saline (TURis) system (Olympus), Vista Coblation/CTR (controlled tissue resection) system (ACMI), Karl Storz, and Wolf^5^. Of these, plasmakinetic resection of the prostate (PKRP) is the most mature technology, showing an improved safety profile^6^. Whether PKRP will replace TURP and become a new standard surgical procedure for the treatment of BPH remains unclear. Currently, there are many published randomized controlled trials (RCTs). In order to provide more definite evidence on this issue, we performed this systematic review.

## Methods

This review was conducted according to the recommendations of the Cochrane Collaboration and followed the Preferred Reporting Items for Systematic Reviews and Meta-Analyses (PRISMA) statement^7^. The protocol of this systematic review is registered in PROSPERO: International prospective register of systematic review (registration number: CRD42014007392)^8^.

### Eligibility criteria

According to the principle of PICOS (participant, intervention, comparison, outcomes, and study design)^7^, the following criteria were used for study selection:

- Participants: BPH patients (any race and nationality) who required surgical treatment, but excluded patients with co-existing neurogenic bladder, unstable bladder, preoperative urethral stricture, or serious urinary tract infection, or patients with a history of lower urinary tract cancer.
- Intervention: PKRP.
- Comparison: TURP.
- Outcomes:
  - ⚬ efficacy outcomes: International Prostate Symptom Score (IPSS), maximum flow rate (Qmax) (ml/s), quality of life (QoL), post-void residue (PVR) (ml), and the International Index for Erectile Function (IIEF).
  - ⚬ safety outcomes: perioperative indicators (operation time (min), drop in hemoglobin level (g/dl), drop in serum sodium level (mmol/L),catheterization time (hour), hospital stay (day)); intraoperative complications (TUR syndrome, blood transfusion); short-term postoperative complications (clot retention, acute urinary retention/re-catheterization, urinary tract infection/fever); long-term postoperative complications (urethral stricture, bladder neck contracture, re-operation).
- Study design: RCT.

Studies were excluded as follows: (a) full-text articles were unavailable, for which we contacted the original study authors and got no response; (b) important information was missing and we were unable to obtain further data from the study authors; (c) when two studies from the same institution reported a similar follow-up interval and the same results, we included the study with better quality and/or more comprehensive information, and contacted the first author to clarify the difference.

### Information sources and search strategies

The relevant published studies were systematically searched from PubMed, ISI Web of Knowledge, Embase, and the Cochrane Library up to September 30, 2013 (search updated on April 10, 2014). The search strategies were provided in Supplementary Information. No regional, publication status, or language restriction was applied. In addition, we screened reference lists of relevant review articles and reports of included studies for further potentially relevant studies. Two authors independently conducted literature search and results were cross-checked.

### Data extraction and methodological quality assessment

Three authors independently screened the studies, read the full texts, and extracted the following data from included studies using a pre-standardized data extraction form: study inclusion criteria and sample size, methods of sampling and grouping, types of participants, interventions/comparisons, outcome measures, follow-up duration, loss-to-follow-up rates and reasons for losses, and statistical methods of the studies. In cases of missing data, we made attempts to contact the study investigators for further information or estimated them if usable data were available. For continuous variables, the standard deviations (SDs) were estimated based on the sample size, range, and median^9^ or *p* value and the sample size^10^; SDs of absolute changes from baseline were imputed and we used a correlation of r = 0.5 as described in the Cochrane Handbook for Systematic Reviews of Interventions^10^. For binary variables, 0.5 into zero cells were added in meta-analyses^11^.

The methodological quality of included studies was assessed using the Cochrane collaboration’s tool for assessing risk of bias^10^. The tool contains seven aspects: (a) randomization method, (b) concealment of allocation, (c) blinding of outcome assessors, (d) blinding of study personnel and participants, (e) incomplete outcome data, (f) selective outcome reporting, and (g) other sources of bias.

### Data analysis

STATA version 12.0 (Stata Corp) was used for statistical analysis. Pooled relative risks (RRs), weighted mean differences (WMDs) and the corresponding 95% confidence intervals (CIs) were calculated. Heterogeneity was quantified by the *I*^2^ statistic^12^, with *I*^2^ values of 40%, 70%, and 100% representing low, moderate, and high heterogeneity, respectively^13^. The fixed-effect model was used when the *I*^2^ value was 40% or lower^10^; otherwise the random-effects model was used. To explore possible sources of heterogeneity, subgroup and sensitivity analyses were performed^14^. In order to test the robustness of the main results, we also investigated the influence of single study by sequentially removing each study^14^. Potential publication bias was assessed by the Egger’s test.

Trial sequential analysis (TSA) was based on the quantification of the required information size. We assessed the required information size (RIS) adjusted for diversity since the heterogeneity adjustment with *I*^2^ might undervalue the required information size. The TSA was performed to maintain an overall 5% risk of a type I error and 20% of the type II error (a power of 80%)^15^,^16^,^17^,^18^. This analysis was conducted according to the previous meta-analysis^18^.

## Results

### Results of search and characteristics of included studies

**Figure 1:**
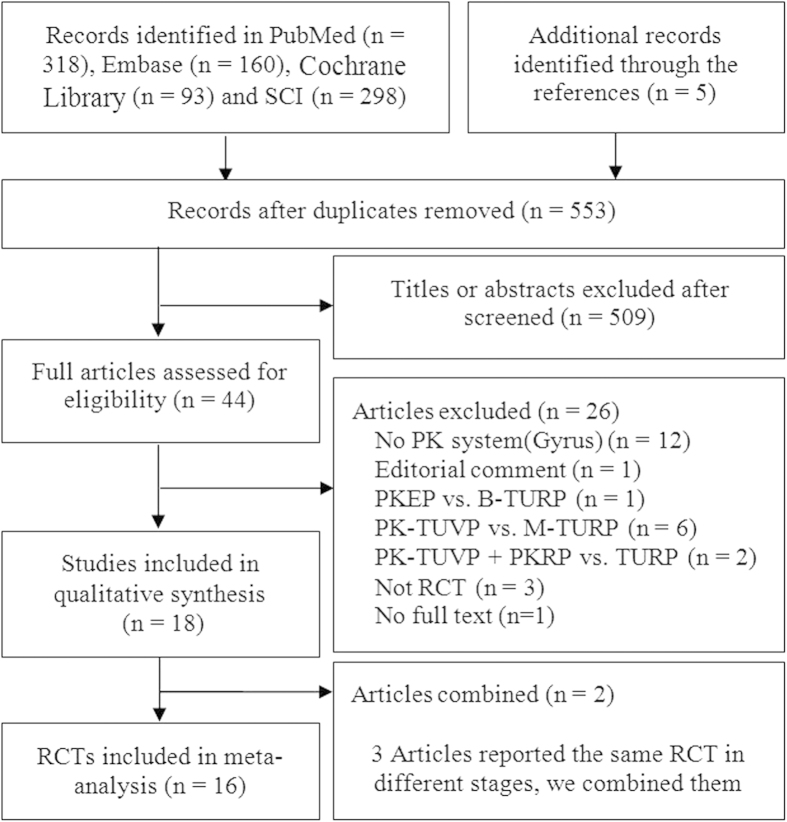
Flow-chart of selecting RCTs for analysis.

The initial search yielded a total of 874 reports. This study selection process is illustrated in a PRISMA flow diagram (Fig. 1). As a result, 18 reports^19^,^20^,^21^,^22^,^23^,^24^,^25^,^26^,^27^,^28^,^29^,^30^,^31^,^32^,^33^,^34^,^35^,^36^ describing 16 RCTs^19^,^20^,^22^,^23^,^24^,^25^,^27^,^28^,^29^,^30^,^31^,^32^,^33^,^34^,^35^,^36^ enrolling 1645 participants were eventually included. Characteristics of included studies and findings of their assessment of risk of bias are summarized in Tables 1 and 2. All the 16 included studies used the Plasmakinetic system^19^,^20^,^22^,^23^,^24^,^25^,^27^,^28^,^29^,^30^,^31^,^32^,^33^,^34^,^35^,^36^. The baselines of them were similar. Besides, a number of studies did not report the required outcome indicators and we thereby estimated the standard deviations^25^,^29^,^32^,^35^,^36^.

### Efficacy outcomes

#### IPSS

Data on IPSS at 3, 6, 12, 24 and 36 months were reported. Three trials^25^,^32^,^36^ reported IPSS at 3 months, four at 6 months^22^,^25^,^32^,^36^, and eight trials reported IPSS at 12 months^23^,^25^,^27^,^28^,^31^,^32^,^34^,^36^. Meta-analysis using a random-effects indicated that there was no statistically significant difference in IPSS at 3, 6, and 12 months (95% CI, –0.37 to 0.31, –0.27 to 0.24, and –0.36 to 0.05, respectively). The mean IPSS at 24 and 36 months, reported by only one trial^34^, favored the PKRP group (95% CI, –1.87 to –0.75, and –2.18 to –1.12, respectively) (Fig. S1).

#### Qmax

**Figure 2:**
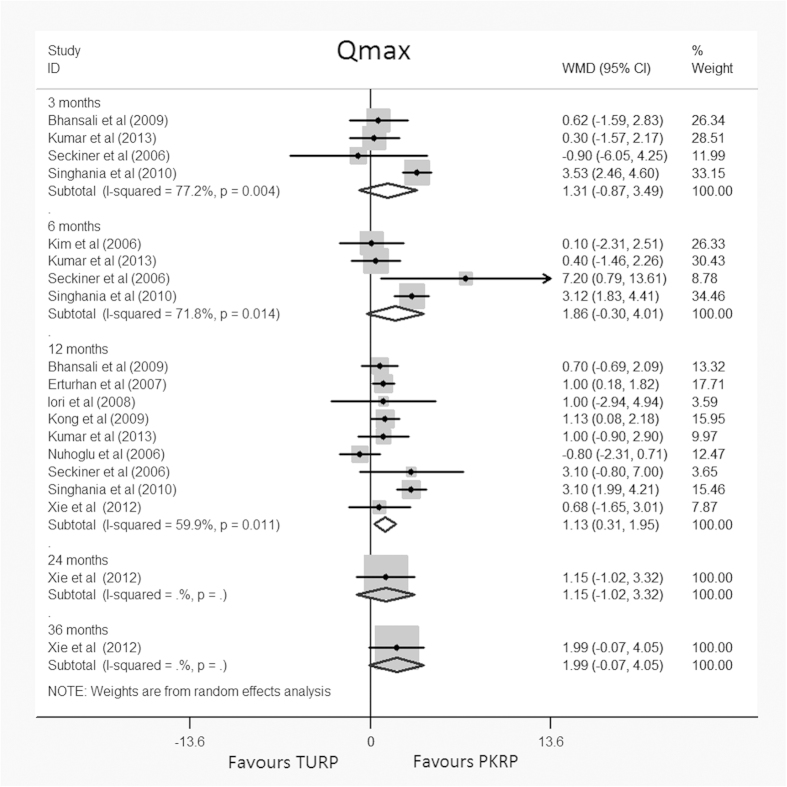
Forest plots for maxium flow rate (Qmax) at 3, 6, 12, 24, 36 momonths of follow up. WMD = weight mean difference; CI = confidence interval. Random effects model used.

Data on Qmax were available at 3, 6, 12, 24 and 36 months. Four studies reported Qmax at 3 months^25^,^30^,^32^,^36^, four at 6 months^22^,^25^,^32^,^36^, nine at 12 months^23^,^25^,^27^,^28^,^30^,^31^,^32^,^34^,^36^, with one study reporting data at 24 and 36 months^34^. This meta-analysis found no significant differences between the groups at 3, 6, 24 and 36 months (95% CI, –0.87 to 3.49, –0.30 to 4.01, –1.02 to 3.32, and –0.07 to 4.05, respectively); however, a significant difference favoring PKRP at 12 months (WMD: 1.13 ml/s, 95% CI, 0.31 to 1.95) was found (Fig. 2). TSA at 12 months showed that there was insufficient evidence to support a reduction of 0.5 ml/s without crossing the trial sequential alpha spending monitoring boundary (TSA adjusted 95% CI, –0.67 to 3.07) (Fig. S2).

#### QoL

The QoL was reported at 3, 6, 12, 24 and 36 months. Two trials at 3 months^25^,^36^, two at 6 months^25^,^36^ and six trials reported QoL data at 12 months^25^,^27^,^28^,^31^,^34^,^36^. Random-effects meta-analyses found no significant differences between PKRP and TURP groups (95% CI, –0.35 to 0.10, –0.22 to 0.22, and –0.27 to 0.17, respectively) (Fig. S3), One study reported quality of life findings at 24 and 36 months^34^, results of which favored the PKRP group (95% CI, –0.62 to –0.26, and –0.48 to –0.18, respectively).

#### PVR

Data on PVR were available at 3, 6, 12, 24 and 36 months. One trial^36^ reported data at 3 months, one trial^36^ at 6 months, and 6 trials^23^,^27^,^28^,^31^,^34^,^36^ at 12 months. Meta-analysis results showed no statistically significant differences in PVR at 3, 6 and 12 months (95% CI, –1.38 to 7.88, –1.04 to 9.58, and –14.12 to 0.12, respectively). Only one study gave mean PVR at 24 and 36 months^34^, which favored the PKRP group (95% CI, –5.32 to –2.14, and –1.95 to – 0.25, respectively) (Fig. S4).

#### IIEF

Three trials^19^,^35^,^36^ reported IIEF data but since the SDs were not given, we were unable to estimate usable data and meta-analysis was not possible. Individual trial results found no significant differences between PKRP and TURP groups.

### Safety outcomes

#### Perioperative outcomes

Pooling results from 10 trials^20^,^22^,^23^,^24^,^25^,^27^,^33^,^34^,^35^,^36^ which assessed the operating time (min) revealed no significant difference between PKRP and TURP (WMD: –3.13 min, 95% CI, –8.10 to 1.84, Fig. S5).

Nine trials^22^,^25^,^29^,^31^,^32^,^33^,^34^,^35^,^36^ reporting postoperative change in hemoglobin level were pooled by a random-effects meta-analytical model. Results showed a combined WMD of –0.66 g/dl (95% CI, –1.38 to 0.06, Fig. S6). TSA adjusted 95% CI was –3.59 to 2.27 g/dl. TSA showed that insufficient evidence was available to show a reduction of 0.5 g/dl without crossing of the trial sequential alpha spending monitoring boundary (Fig. S7).

Six trials^22^,^29^,^31^,^32^,^33^,^34^ reporting postoperative drop in serum sodium level were pooled using random-effect model in the meta-analysis. The result of analysis showed a significantly lower drop after PKRP approach (WMD: –2.02 mmol/L, 95% CI, –3.35 to –0.69, Fig. S8). TSA adjusted 95% CI was –7.42 to 4.29 mmol/L. TSA demonstrated that insufficient evidence was available to show a reduction of 1 mmol/L, without crossing of the trial sequential alpha spending monitoring boundary (Fig. S9). Eleven trials^22^,^23^,^24^,^25^,^27^,^28^,^30^,^31^,^34^,^35^,^36^ reporting mean catheterization time were pooled by random-effect model in the meta-analysis. The result of analysis revealed a significantly shorter catheterization time in the PKRP group (WMD: –19.66 h, 95% CI, –26.56 to –12.77, Fig. S10). TSA adjusted 95% CI was –47.80 to 8.47 h. TSA displayed that evidence which was available to show a reduction of 5 h was insufficient, without crossing of the trial sequential alpha spending monitoring boundary (Fig. S11).

Seven trials^22^,^27^,^28^,^30^,^31^,^34^,^35^ reported mean hospital stay and were pooled with random-effect model. The result of pooled data revealed a significantly shorter hospital stay in the PKRP group (WMD: –0.85 d, 95% CI, –1.44 to –0.27, Fig. S12). TSA adjusted 95% CI was –2.74 to 1.04 d. TSA revealed that insufficient evidence was available to show a reduction of 0.5 d, without crossing of the trial sequential alpha spending monitoring boundary (Fig. S13).

#### Specific intraoperative complications

**Figure 3:**
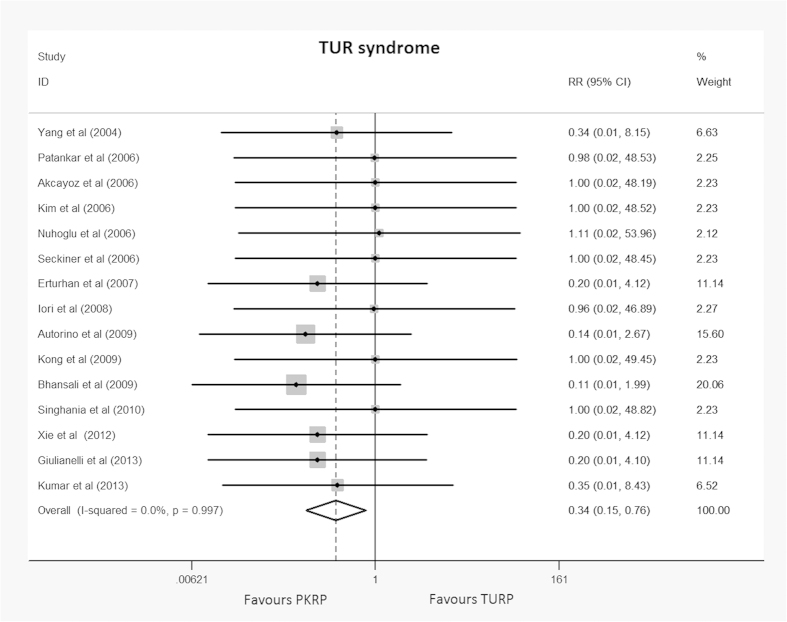
Forest plot of TUR syndrome. RR = relative risk; CI = confidence interval. Fixed effects model used.

**Figure 4:**
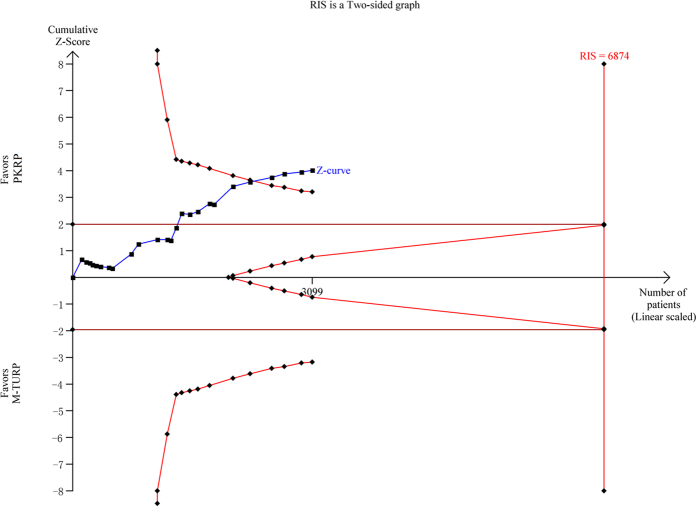
Trial sequential analysis of TUR syndrom. A diversity adjusted information size of 6874 patients was calculated using a two side α = 5%, β = 20% (power 80%), D^2^ = 0%, an anticipated relative risk increase of 35% and an event propotion of 2% in the control arm. Trials with no events were included in the study with a constant continuity correction of 1.

This meta-analysis of TUR syndrome included data from a total of 15 trials^19^,^20^,^22^,^23^,^24^,^25^,^27^,^28^,^29^,^30^,^31^,^32^,^34^,^35^,^36^. This complication occurred in 15 of the 757 patients undergoing TURP (2.0%), and none of the 742 participants undergoing PKRP (0%). The difference was statistically significant (RR: 0.34, 95% CI, 0.15 to 0.76). Detailed results are summarized in Fig. 3. A constant continuity correction 1.0 was applied for zero-event trials. TSA showed that 3099 (45%) of the required information size of 6874 was accrued to detect or reject a 35% reduction in relative risk, and the cumulative Z-curve crossed the conventional boundary for favoring PKRP without crossing of the trial sequential alpha spending monitoring boundary indicating sufficient evidence favoring PKRP in terms of TUR syndrome (TSA adjusted 95% CI, 0.01 to 0.77, Fig. 4).

**Figure 5:**
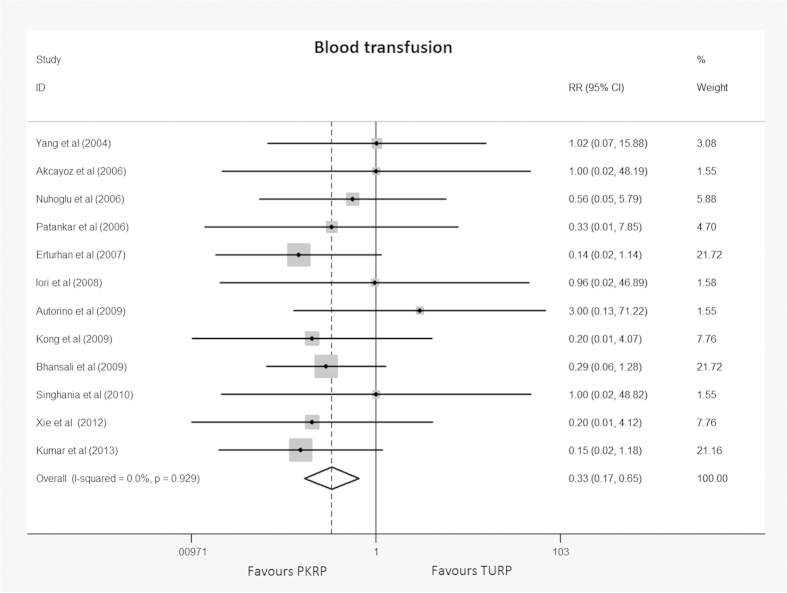
Forest plot of cases requiring blood transfusion. RR = relative risk; CI = confidence interval. Fixed effects model used.

**Figure 6:**
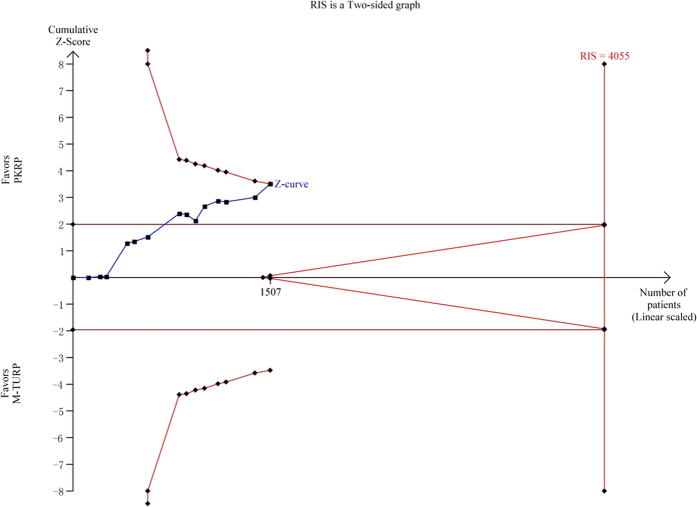
Trial sequential analysis of blood transfusion. A diversity adjusted information size of 4423 patients was calculated using a two side α = 5%, β = 20% (power 80%), D^2^ = 0%, an anticipated relative risk increase of 35% and an event propotion of 5% in the control arm. Trials with no events were included in the study with a constant continuity correction of 1.

Twelve trials^19^,^20^,^23^,^24^,^27^,^28^,^29^,^30^,^31^,^32^,^34^,^36^ reporting blood transfusion were included in the meta-analysis. In all, 7 of 616 participants undergoing PKRP and 29 of 599 undergoing TURP required blood transfusion with an RR of 0.33 (95% CI 0.17 to 0.65), and the result was statistically significant (*p* < 0.001). Detailed results were summarized in Fig. 5. Applying a constant continuity correction of 1 for no-event trials did not change the results significantly. TSA presented that 1507 (37%) of required information size of 4055 patients was accrued to detect or reject a 35% diminution in relative risk, but the cumulative Z-curve crossed the conventional boundary for favoring PKRP and surpassed the trial sequential alpha spending monitoring boundary indicating firm evidence favoring PKRP in terms of blood transfusion (TSA adjusted 95% CI, 0.10 to 0.99, Fig. 6).

#### Short-term postoperative complications

**Figure 7:**
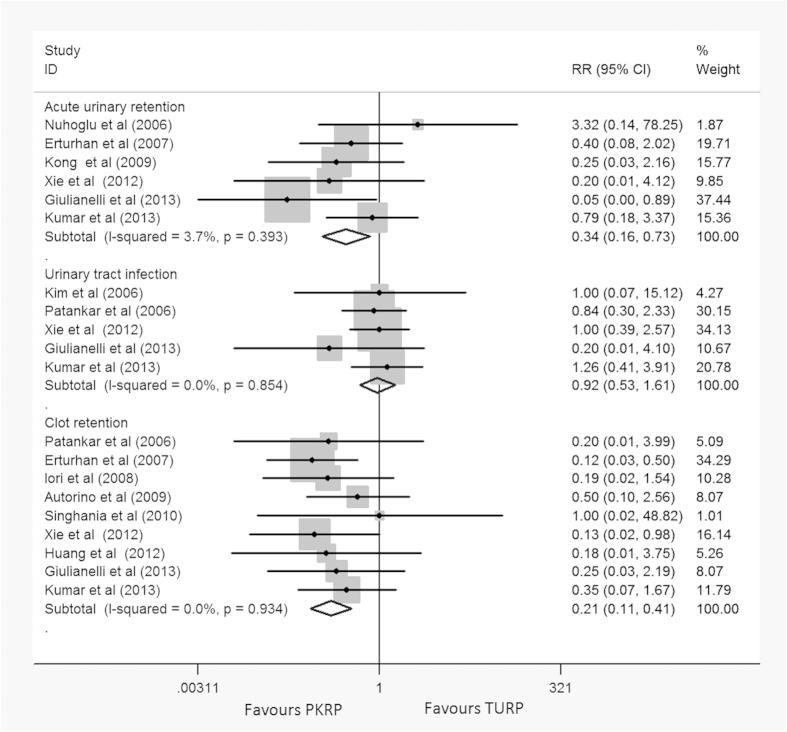
Forest plots of short-term postoperative complications. RR = relative risk; CI = confidence interval. Fixed effects model used.

**Figure 8:**
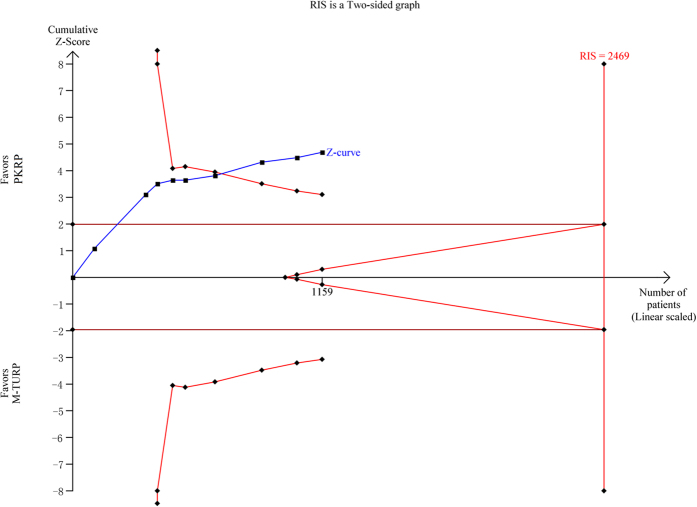
Trial sequential analysis of clot retention. A diversity adjusted information size of 2469 patients was calculated using a two side α = 5%, β = 20% (power 80%), D^2^ = 0%, an anticipated relative risk increase of 35% and an event propotion of 9% in the control arm. Trials with no events were included in the study with a constant continuity correction of 1.

Meta-analysis of 9 trials^24^,^27^,^28^,^29^,^32^,^33^,^34^,^35^,^36^ using a fixed-effect model (I^2^ = 0%) showed that the clot retention rate was reduced in PKRP group, and there was significant difference between them (RR: 0.21, 95% CI, 0.11 to 0.41, Fig. 7). TSA demonstrated that 1159 (47%) of required information size of 2469 patients was accrued to detect or reject a 35% diminution in relative risk, however, the cumulative Z-curve crossed the conventional boundary for favoring PKRP providing firm evidence of more safety in clot retention treated by PKRP compared to TURP (TSA adjusted 95% CI, 0.07 to 0.58, Fig. 8).

Meta-analysis of 6 trials^23^,^27^,^31^,^34^,^35^,^36^ by a fixed-effects model (I^2^ = 30.4%) showed fewer acute urinary retention/re-catheterization need in the PKRP group, and there was significant difference between them (RR: 0.34, 95% CI, 0.16 to 0.73, Fig. 7).

Meta-analysis of 5 trials^22^,^24^,^34^,^35^,^36^ by a fixed-effect model (I^2^ = 0%) found no significant difference between two groups in urinary tract infection (RR: 0.92, 95% CI, 0.53 to 1.61) (Fig. 7).

#### Long-term postoperative complications

Data from 7 trials^23^,^27^,^28^,^29^,^34^,^35^,^36^ that assessed bladder neck contracture revealed significant difference between the two groups, favoring PKRP (RR: 0.46, 95% CI, 0.23 to 0.94, Fig. S14).

Seven trials^22^,^23^,^25^,^27^,^29^,^34^,^36^ reported urethral stricture, three trials^27^,^29^,^34^ reported re-operation rate. they all showed no significant difference between PKRP and TURP (Fig. S14).

### Sensitivity analysis and investigation of publication bias

Sensitivity analysis was performed by removing each study sequentially. According to the results, no significant changes were observed for pooled RRs or WMDs and relevant 95% CIs for the whole process, suggesting that all the pooled results were not influenced by any included single study and the results of this meta-analysis were stable. Publication bias as assessed by the Egger’s test indicated that only the analysis of TUR syndrome demonstrated significant publication bias (*p* < 0.001).

## Discussion

### Major findings

This study was based on 16 RCTs^19^,^20^,^22^,^23^,^24^,^25^,^27^,^28^,^29^,^30^,^31^,^32^,^33^,^34^,^35^,^36^ comparing the efficacy and safety of PKRP and TURP for BPH with a total of 1645 patients. All trials were of low or moderate risk of bias. The results indicated that the curative effects of PKRP and TURP were similar and both of them significantly improved symptoms in patients with BPH. In addition, This meta-analysis also showed that PKRP was better than TURP in terms of Qmax improvement at 12 months. However, it was indeterminate as to long-term follow-up (>12 months) results, because we could not get reliable results due to limited size of included studies. Hence, we were only able to infer that PKRP may have potential advantage in the curative effect.

For surgical blood loss, blood transfusion, and clot retention, PKRP was manifestly better than TURP. It may be associated with better plasmakinetic bipolar coagulation technology^37^. TSA provided firm evidence of reduced blood transfusion and clot retention rate in PKRP group as compared with TURP group.

For TUR syndrome and drop in serum sodium level, PKRP was also better than TURP. We even found that incidence rate of TUR syndrome was 0% in PKRP group. The reason may be that plasmakinetic bipolar technology was completed under saline flushing, which may help avoid loss of serum sodium and prevent TUR syndrome. TSA also suggested sufficient evidence of enhanced safety with PKRP in terms of TUR syndrome.

For perioperative indicators, PKRP was associated with shorter catheterization time and hospital stay. This may be explained by the fact that plasmakinetic bipolar coagulation technology has greater hemostatic capacity than traditional TURP. However, there was no significant difference with respect to operation time.

For short-term and long-term postoperative complications, there was no significant difference in urinary tract infection, urethral stricture and re-operation.

### Strengths and limitations

This systematic review has several strengths. Firstly, when we searched the Cochrane Library, a relevant Cochrane protocol by Liu *et al.*^38^ was found, which has been withdrawn due to a lack of progress. We thought it was very meaningful to complete this subject and this study was performed. Secondly, We are aware of four relevant published systematic reviews and meta-analyses^6^,^39^,^40^,^41^, all of which focused on comparing efficacy and safety of monopolar TURP and B-TURP and included smaller number of studies and outcome measures than ours. Compared to other bipolar resection equipment, the PK system is used more frequently and this technology is more mature. Therefore, we believe that the different bipolar devices are also likely to influence the outcomes of the study. Thirdly, this study is based on a published protocol with rigid, pre-defined inclusion criteria^8^. No restrictions on language or outcome reporting were applied during our comprehensive literature search. In addition, the strength of available evidences was assessed by conducting subgroup analysis according to the level of risk of bias in included studies, and performed trial sequential analysis for all statistically significant outcomes^18^,^42^. To our knowledge, this is the first time applied trial sequential analysis for this topic.

The limitations of this analysis are reflected by the fundamental weaknesses of the included trials. Firstly, almost all the included studies were assessed as having moderate risk of bias (table 2), therefore, our results should be interpreted with caution^43^,^44^. Secondly, due to the lack of dada, subgroup analyses could not be performed by patients’ age, race, and prostate size, and this might influence the extrapolation of the results. Thirdly, data are also sparse for certain long-term outcomes, such as curative effect at 2-year or 3-year. Fourthly, no enough data on outcomes of sexual function and cost-effect were available. Lastly, for some late postoperative complications (retrograde ejaculation, re-operation), the study sample size and overall sample size are small.

#### Implications for practice and research

This study has some implications for clinical practice and further research. Further researches should clarify the safety, effectiveness, potential advantages and disadvantages of PKRP compared with TURP in large multicenter RCTs, covering outcomes related to sexual functions, cost-effect and long-term outcomes. In clinical practice, surgeons should not be limited to choose monopole TURP to treat BPH. Although TURP was still considered as the “standard procedure” in guidelines, this research indicated that PKRP had advantage on operation safety. Therefore, PKRP may be a better choice, especially for old patients and those with large volume prostate or high risk disease.

## Conclusion

This meta-analysis indicates that PKRP may be associated with reduced blood loss, reduced blood transfusion and clot retention rate, shorter catheterization time and hospital stay, and absence of TUR syndrome. TSA provided firm evidence of improved safety profile in terms of blood transfusion and clot retention. Moreover, curative effects of PKRP and TURP are non-inferior to each other, with PKRP showing potential additional benefits. PKRP poses as a viable alternative to TURP as a new standard surgical procedure. Data from well-conducted multicenter RCTs with large sample size and long-term follow-up (>12 months) with additional analyses of cost-effectiveness and sexual function are warranted.

## Additional Information

**How to cite this article**: Li, S. *et al.* Plasmakinetic resection technology for the treatment of benign prostatic hyperplasia: evidence from a systematic review and meta-analysis. *Sci. Rep.* **5**, 12002; doi: 10.1038/srep12002 (2015).

## Supplementary Material

Supplementary Information

## Figures and Tables

**Table 1:** Characteristics of included studies.

Authors	Year	Bipolarequipment	No ofpatients	Max. followup (months)	Age(years)	Prostatevolume(ml)	IPSS	QoL	Qmax(ml/s)	PVR (ml)	IIEF
Yang *et al.*^19^	2004	PK	58/59	3	NA	45.8/48.9	20.9/21.6	3.7/4.0	10.4/10.9	99.0/150.0	8.2/7.8
Akcayoz *et al.*^20^	2006	PK	21/21	NA	67.0/66.0	40.0/47.0	NA	NA	NA	NA	NA
Kim *et al.*^22^	2006	PK	25/25	6	68.1/70.6	51.7/53.2	19.0/18.6	NA	6.5/6.1	NA	NA
Nuhoglu *et al.*^23^	2006	PK	27/30	12	64.6/65.2	47.0/49.0	17.6/17.3	NA	6.9/7.3	96.0/88.0	NA
Patankar *et al.*^24^	2006	PK	52/51	1	64.0/62.0	51.3/52.3	23.3/23.7	NA	5.9/6.4	NA	NA
Seckiner *et al.*^25^	2006	PK	24/24	12	61.2/63.9	49.4/41.4	24.1/23.2	NA	8.5/8.3	NA	NA
Erturhan *et al.*^27^	2007	PK	120/120	12	68.5/67.4	43.0/42.0	23.0/24.0	2.0/3.0	10.9/9.2	NA	NA
Iori *et al.*^28^	2008	PK	27/26	12	65.0/63.0	49.0/48.0	21.0/20.0	3.0/3.6	7.0/8.7	99.0/96.0	NA
Kong *et al.*^31^	2009	PK	51/51	12	68.4/68.5	41.8/43.1	23.3/23.9	4.5/4.5	4.99/4.60	107.0/103.0	NA
Bhansali *et al.*^30^	2009	PK	35/35	9	NA	78.5/78.7	NA	NA	4.4/4.2	NA	NA
Autorino *et al.*^21^,^26^,^29^	2009	PK	35/35	48	61.0/59.0	47.5/51.6	24.3/24.2	3.9/4.2	6.2/7.1	75.0/80.0	NA
Singhania *et al.*^32^	2010	PK	30/30	12	63.9/65.9	40–80	24.1/23.4	NA	6.6/6.4	NA	NA
Xie *et al.*^34^	2012	PK	110/110	60	69.9/64.9	65.9/67.0	23.8/22.8	4.5/4.4	9.8/9.7	94.5/96.3	NA
Huang *et al.*^33^	2012	PK	71/65	NA	65.1/64.6	52.9/50.1	23.4/22.1	4.2/4.1	6.7/6.9	NA	NA
Giulianelli *et al.*^35^	2013	PK	80/80	36	62.5/64.2	47.8/50.0	22.3/23.4	3.3/3.0	8.9/6.5	243.0/187.0	16.0/17.0
Kumar *et al.*^36^	2013	PK	57/60	12	62.3/63.7	50.3/52.2	19.8/20.7	3.6/3.7	7.1/7.0	148.4/139.3	17.3/17.0

PK; Plasmakinetic system, IPSS; International Prostate Symptom Score, Qmax; maximum flow rate, QoL; quality of life, PVR; post-void residue, IIEF; International Index for Erectile Function, NA; not available.

**Table 2:** Risk of bias assessment of included studies.

Trial	Randomsequencegeneration	Allocationconcealment	Blinding ofparticipantsand personnel	Blindingof outcomeassessment	Incompleteoutcomedata	Selectivereporting	Otherbias
Yang *et al.*^19^	Unclear	Unclear	Unclear	Unclear	Low	Low	Low
Akcayoz *et al.*^20^	Unclear	Unclear	Unclear	Unclear	Low	Low	Low
Kim *et al.*^22^	Unclear	Unclear	Unclear	Unclear	Low	Low	Low
Nuhoglu et al^23^	Unclear	Unclear	Unclear	Unclear	Low	Low	Low
Patankar *et al.*^24^	Unclear	Low	Low	Low	Low	Unclear	Low
Seckiner *et al.*^25^	Unclear	Unclear	Unclear	Unclear	Low	Low	Low
Erturhan *et al.*^27^	Unclear	Unclear	Unclear	Unclear	Low	Low	Low
Iori *et al.*^28^	Unclear	Low	Unclear	Unclear	Low	Low	Low
Kong *et al.*^31^	Low	Low	Low	Unclear	Low	Low	Low
Bhansali *et al.*^30^	Low	Low	Unclear	Low	Low	Low	Low
Autorino *et al.*^21^,^26^,^29^	Low	Unclear	Unclear	Low	Low	Low	Low
Singhania *et al.*^32^	Low	Unclear	high	Unclear	Low	Low	Low
Xie *et al.*^34^	Unclear	Low	high	high	Low	Low	Low
Huang *et al.*^33^	Low	Low	Unclear	Unclear	Low	Low	Low
Giulianelli *et al.*^35^	Unclear	Unclear	Unclear	Unclear	Low	Low	Low
Kumar *et al.*^36^	Low	Unclear	Unclear	Unclear	Low	Low	Low
