# Supplementary material for: Plasmakinetic resection technology for the treatment of benign prostatic hyperplasia: evidence from a systematic review and meta-analysis
Source: Sci Rep. 2015 Jul 9;5:12002. doi: 10.1038/srep12002 (PMC4496666; doi:10.1038/srep12002)
Supplement: Supplementary Information [file srep12002-s1.pdf]

The Title: **Plasmakinetic resection technology for the treatment of benign prostatic hyperplasia: evidence from a systematic review and meta-analysis**

The Authors: Sheng Li, Joey S.W. Kwong, Xian-Tao Zeng, Xiao-Lan Ruan, Tong-Zu Liu, Hong Weng, Yi Guo, Chang Xu, Jin-Zhu Yan, Xiang-Yu Meng, Xing-Huan Wang*

Supplementary Information 1

Search strategies

PubMed
(ablative OR “minimally invasive”[tw] OR plasmakinetic* OR plasmasect* OR PKRP OR gyrus OR bipolar) AND (TURP[tw] OR Transurethral prostatectom*[tw] OR Transurethral prostate resection*[tw] OR “Transurethral Resection of Prostate”[Mesh] OR transurethral resection of the prostate[tw]) AND (((((((((((((((((((volunteer*[tw]) OR ((prospectiv*[tw]))) OR ((control*[tw]))) OR ((prospective studies[mh]))) OR ((follow-up studies[mh]))) OR ((evaluation studies[pt]))) OR ((comparative study[pt]))) OR ((research design[mh:noexp]))) OR ((random*[tw]))) OR (((singl* OR doubl* OR trebl* OR tripl*) AND (blind* OR mask*)))) OR ((clinical trial[tw]))) OR ((clinical trials[mh]))) OR ((clinical trial[pt]))) OR ((single-blind method[mh]))) OR ((double blind method[mh]))) OR ((random allocation[mh]))) OR ((randomized clinical trials[mh]))) OR ((controlled clinical trial[pt]))) OR ((randomized controlled trial[pt])))

Embase
(ablative OR minimally invasive OR plasmakinetic* OR plasmasect* OR PKRP OR gyrus OR bipolar) AND ('transurethral resection'/exp OR 'transurethral resection of the prostate' OR 'transurethral prostate resection' OR TURP OR transurethral prostatectom$) AND ('Clinical Trial'/exp OR 'controlled clinical trial'/exp OR random$ OR trial*)

Science Citation Index
TS=(ablative OR minimally invasive OR plasmakinetic* OR plasmasect* OR PKRP OR gyrus OR bipolar) AND TS=(TURP OR Transurethral prostatectom* OR Transurethral prostate resection OR Transurethral Resection of Prostate OR transurethral resection of the prostate) AND TS=(Trial OR trials OR random*)

Cochrane Library
(ablative OR 'minimally invasive' OR plasmakinetic* OR plasmasect* OR PKRP OR gyrus OR bipolar):ti,ab,kw AND (TURP OR Transurethral prostatectom* OR Transurethral prostate resection* OR 'transurethral resection of the prostate'):ti,ab,kw

Supplementary Information 2

Fig S1 Forest plots for International Prostate Symtom Score (IPSS) at 3, 6, 12, 24, 36 months of follow up. WMD=weight mean difference; CI=confidence interval. Random effects model used

Fig S2 Trial sequential analysis of maxium flow rate (Qmax) at 12 months. The required information size for Qmax at 12 months was calculated based on a two side α=5%, β=20% (power 80%), a minimal relevant difference of 0.5 ml/s, a stadard deviation of 28.39 ml/s, and D^2=61% as estimated in a random effects modle

Fig S3 Forest plots for quality of life (QoL) at 3, 6, 12, 24, 36 months of follow up. WMD=weight mean difference; CI=confidence interval. Random effects model used

Fig S4 Forest plots for post-void residual volume urine (PVR) at 3, 6, 12, 24, 36 months of follow up. WMD=weight mean difference; CI=confidence interval. Random effects model used

Fig S5 Forest plot of operative time. WMD=weight mean difference; CI=confidence interval. Random effects model used

Fig S6 Forest plot of hemoglobin decrease. WMD=weight mean difference; CI=confidence interval. Random effects model used

Fig S7 Trial sequential analysis of hemoglobin decrease. The required information size for hemoglobin decrease was calculated based on a two side α=5%, β=20% (power 80%), a minimal relevant difference of 0.5 g/dl, a stadard deviation of 0.92 g/dl, and D^2=98% as estimated in a random effects modle

Fig S8 Forest plot of serum sodium decrease. WMD=weight mean difference; CI=confidence interval. Random effects model used

Fig S9 Trial sequential analysis of serum sodium decrease. The required information size for serum sodium decrease was calculated based on a two side α=5%, β=20% (power 80%), a minimal relevant difference of 1.0 mmol/L, a stadard deviation of 1.57 mmol/L, and D^2=98% as estimated in a random effects modle

Fig S10 Forest plot of time catheter removal. WMD=weight mean difference; CI=confidence interval. Random effects model used

Fig S11 Trial sequential analysis of time catheter removal. The required information size for serum sodium decrease was calculated based on a two side α=5%, β=20% (power 80%), a minimal relevant difference of 5 h, a stadard deviation of 15.23 h, and D^2=97% as estimated in a random effects modle

Fig S12 Forest plot of hospital stay. WMD=weight mean difference; CI=confidence interval. Random effects model used

Fig S13 Trial sequential analysis of hospital stay. The required information size for serum sodium decrease was calculated based on a two side α=5%, β=20% (power 80%), a minimal relevant difference of 0.5 d, a stadard deviation of 0.53 d, and D^2=99% as estimated in a random effects modle

Fig S14 Forest plots of long-term postoperative complications. RR=relative risk; CI=confidence interval. Fixed effects model used

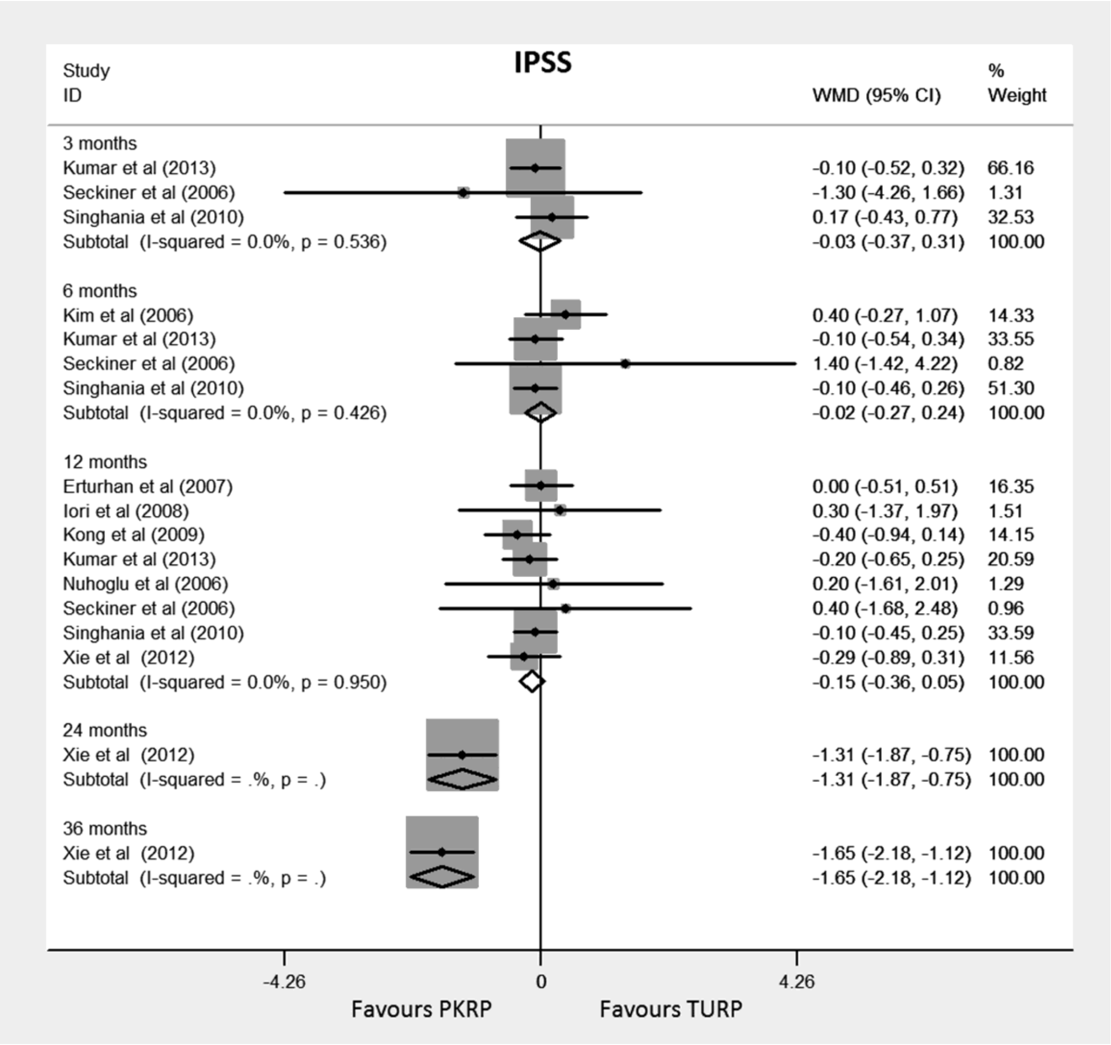

Fig S1 Forest plots for IPSS at 3, 6, 12, 24, 36 mo of follow-up

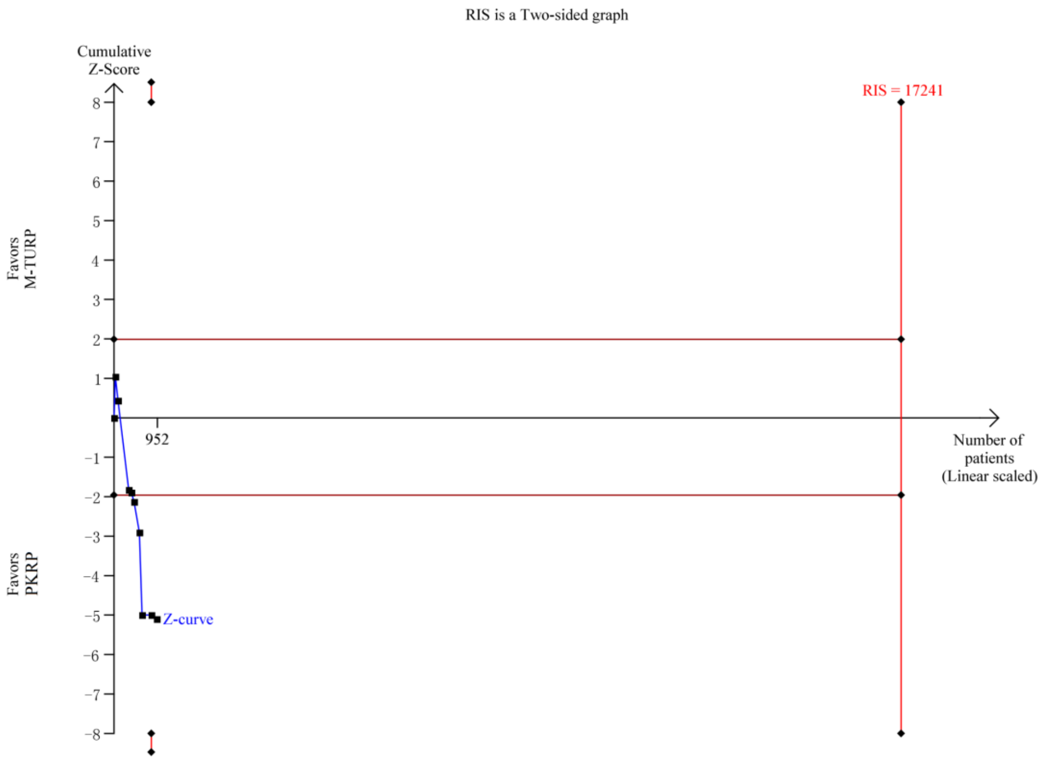

Fig S2 Trial sequential analysis of Qmax at 12 moths

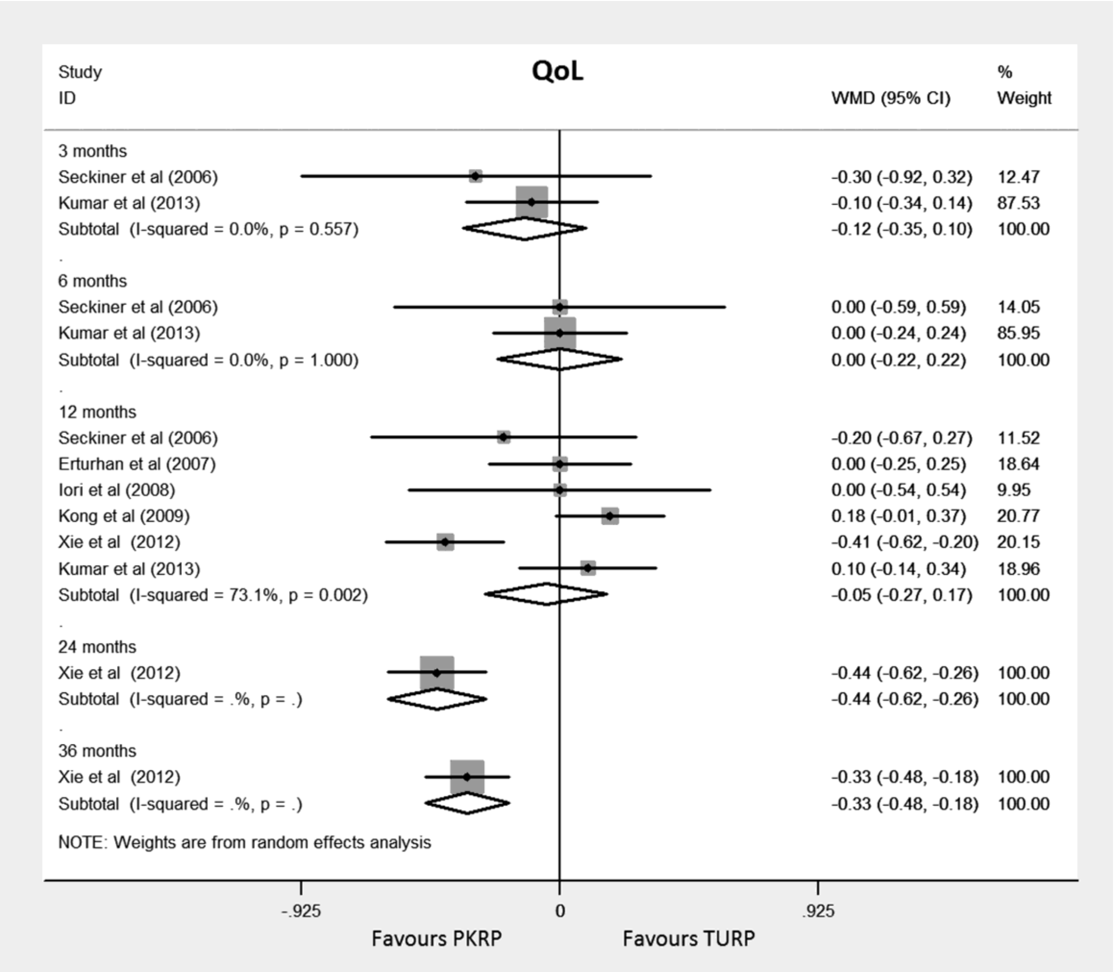

Fig S3 Forest plots for QOL at 3, 6, 12, 24, 36 mo of follow-up

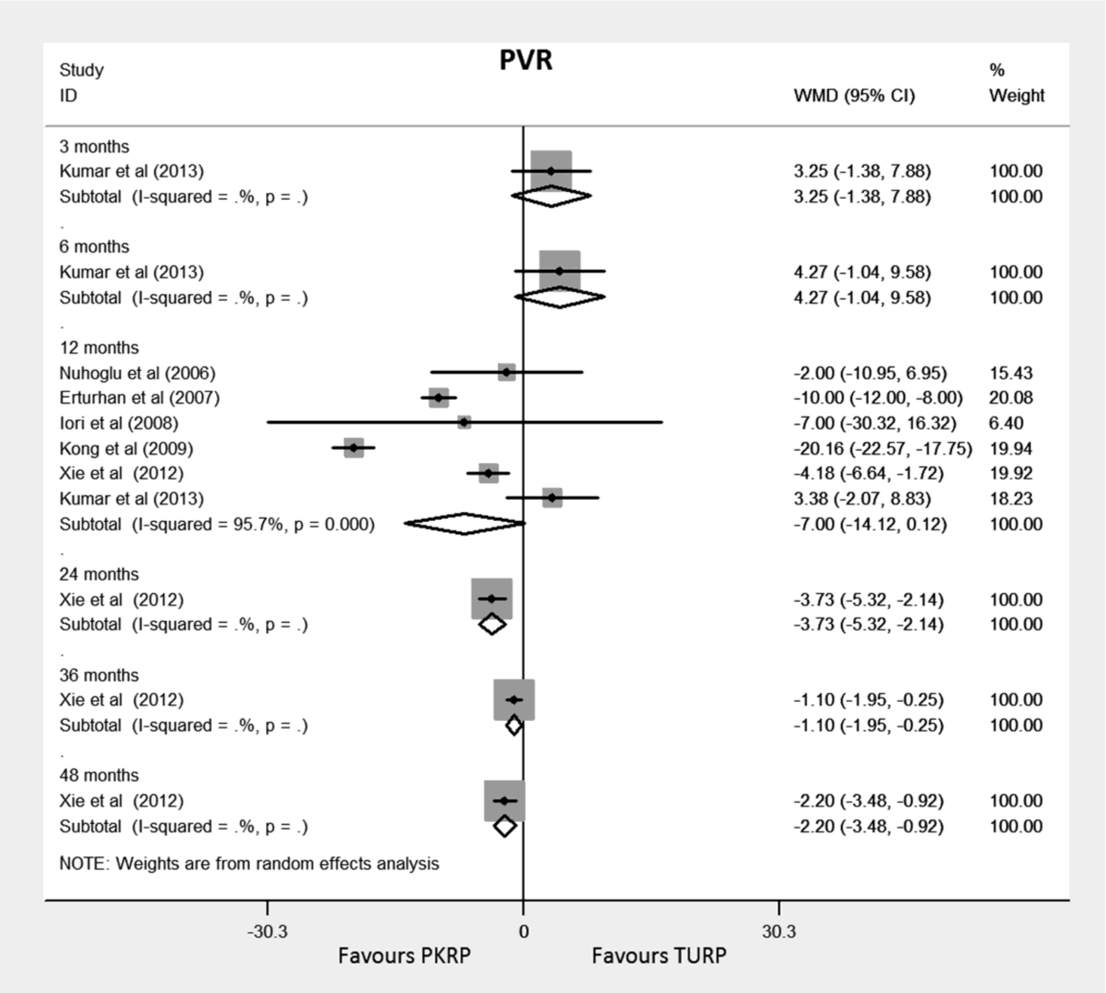

Fig S4 Forest plots for PVR (ml) at 3, 6, 12, 24, 36 mo of follow-up

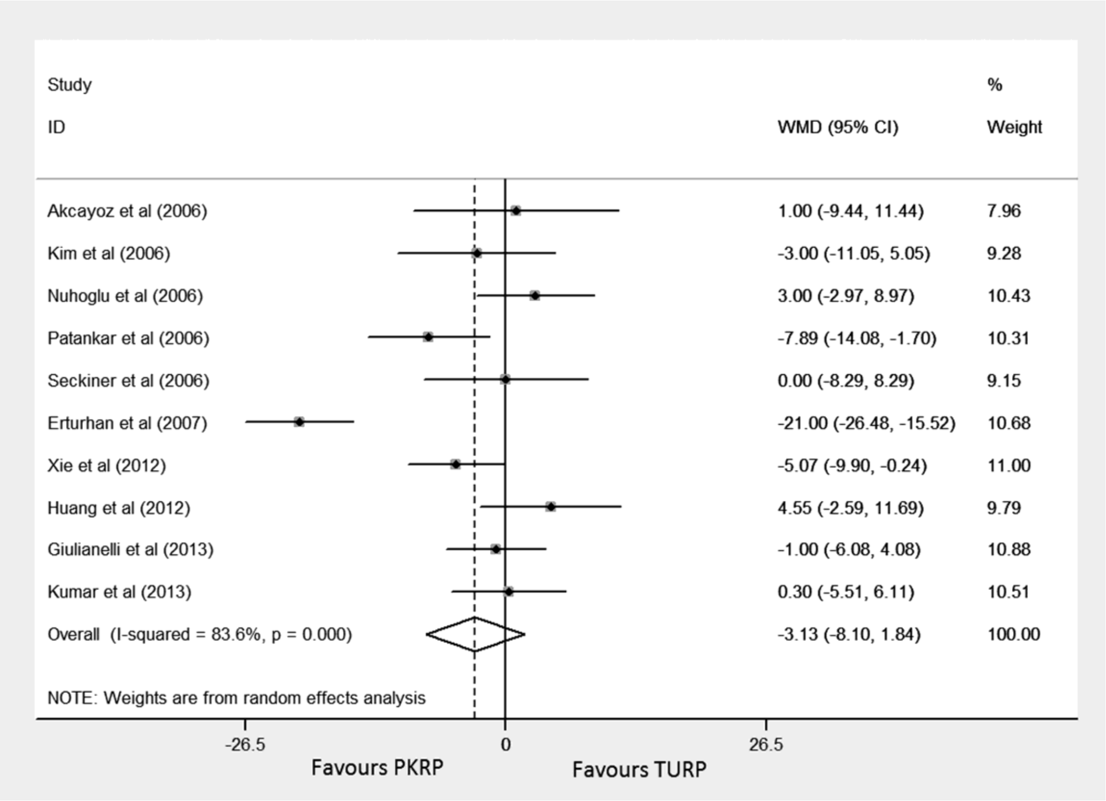

Fig S5 Forest plot of operative time (mins)

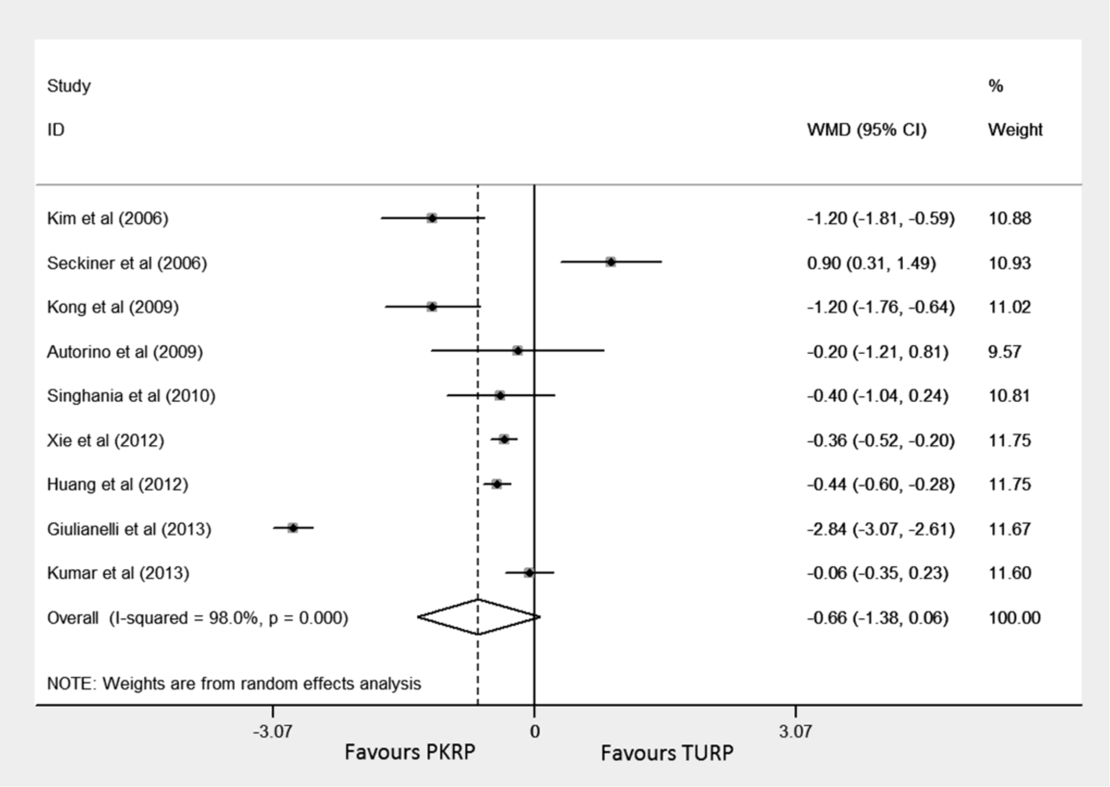

Fig S6 Forest plot of hemoglobin decrease

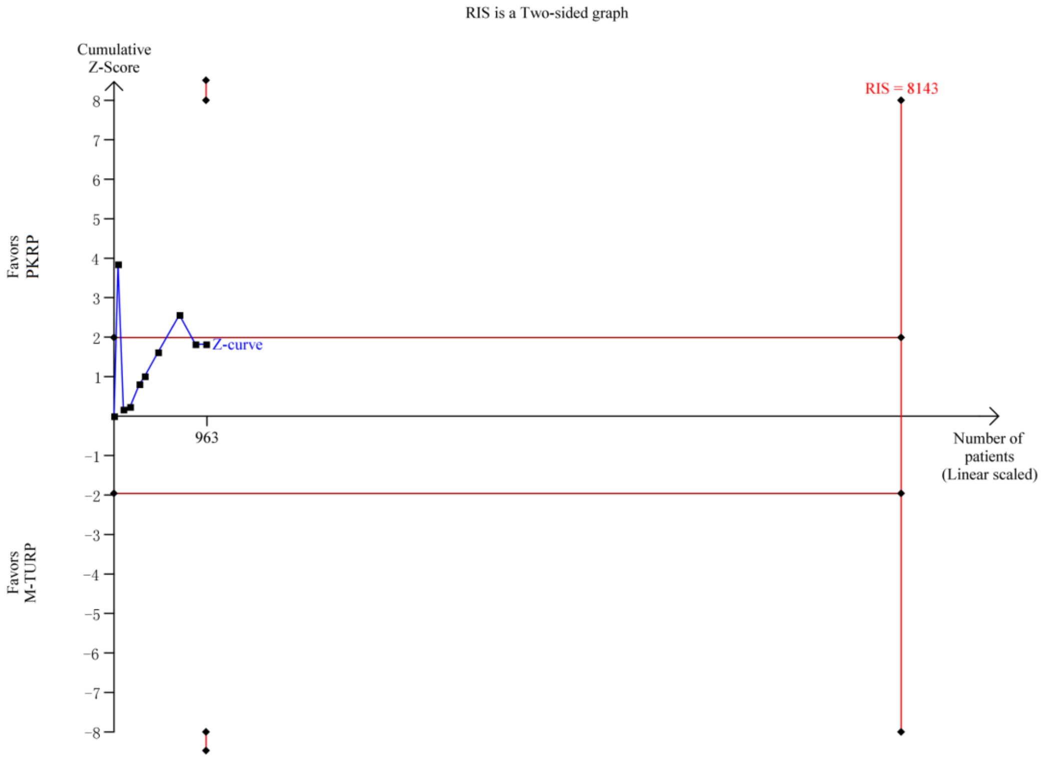

Fig S7 Trial sequential analysis of hemoglobin decrease

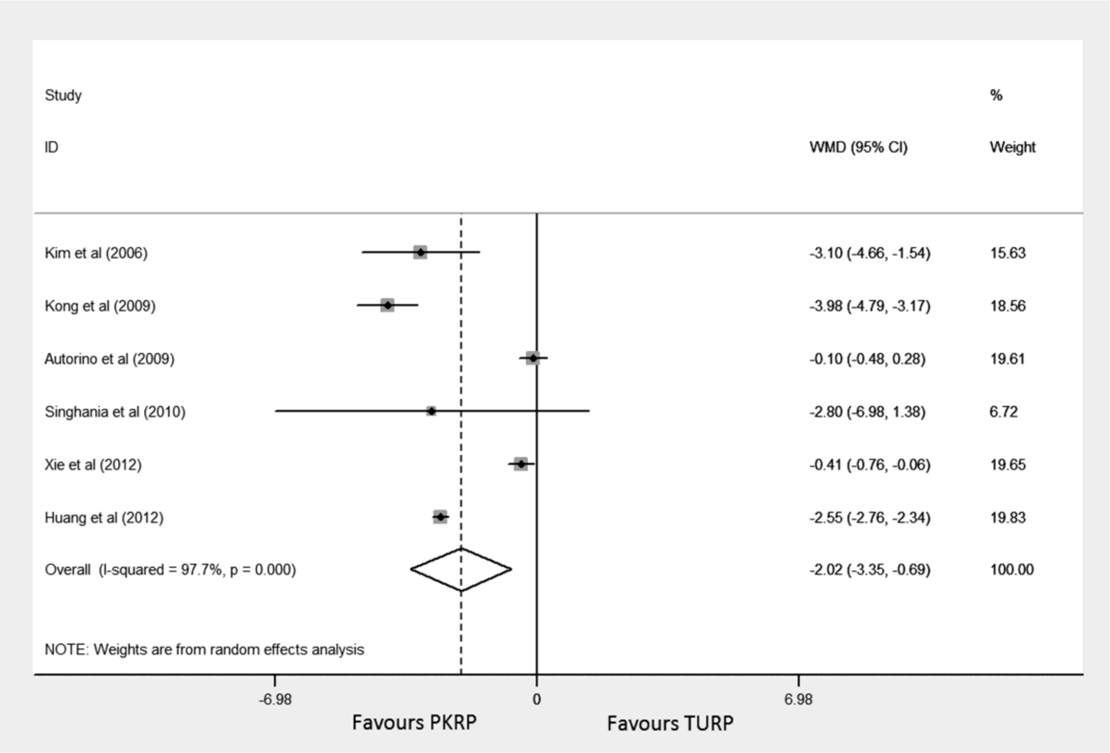

Fig S8 Forest plot of serum sodium decrease

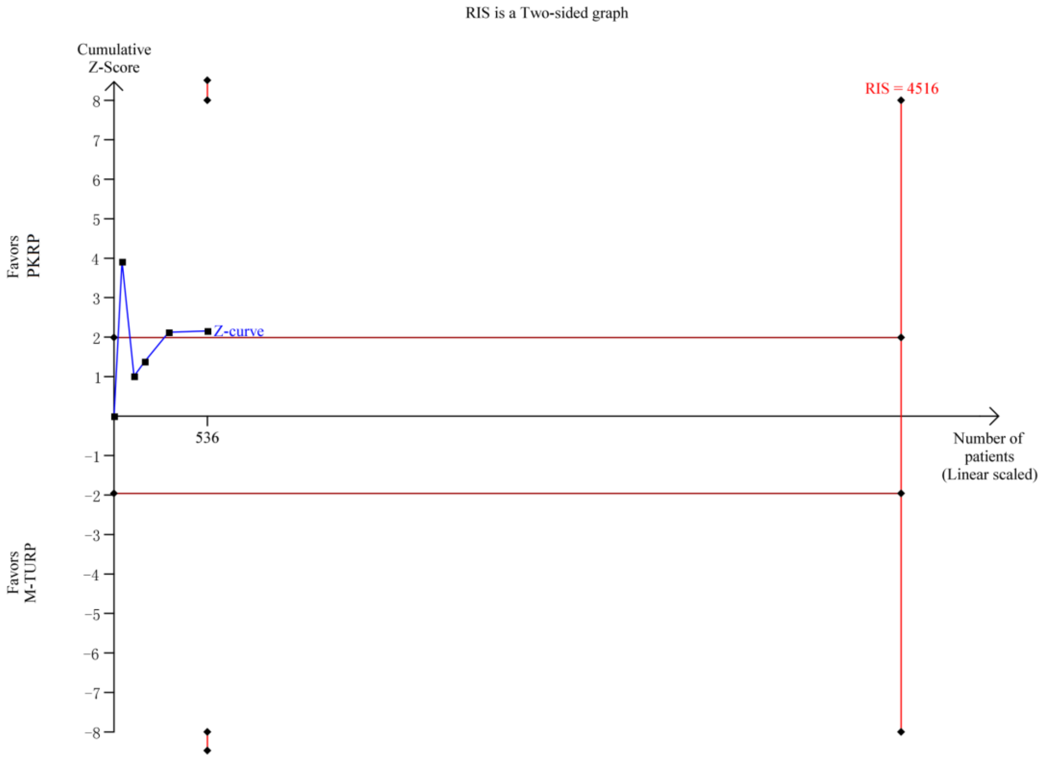

Fig S9 Trial sequential analysis of serum sodium decrease

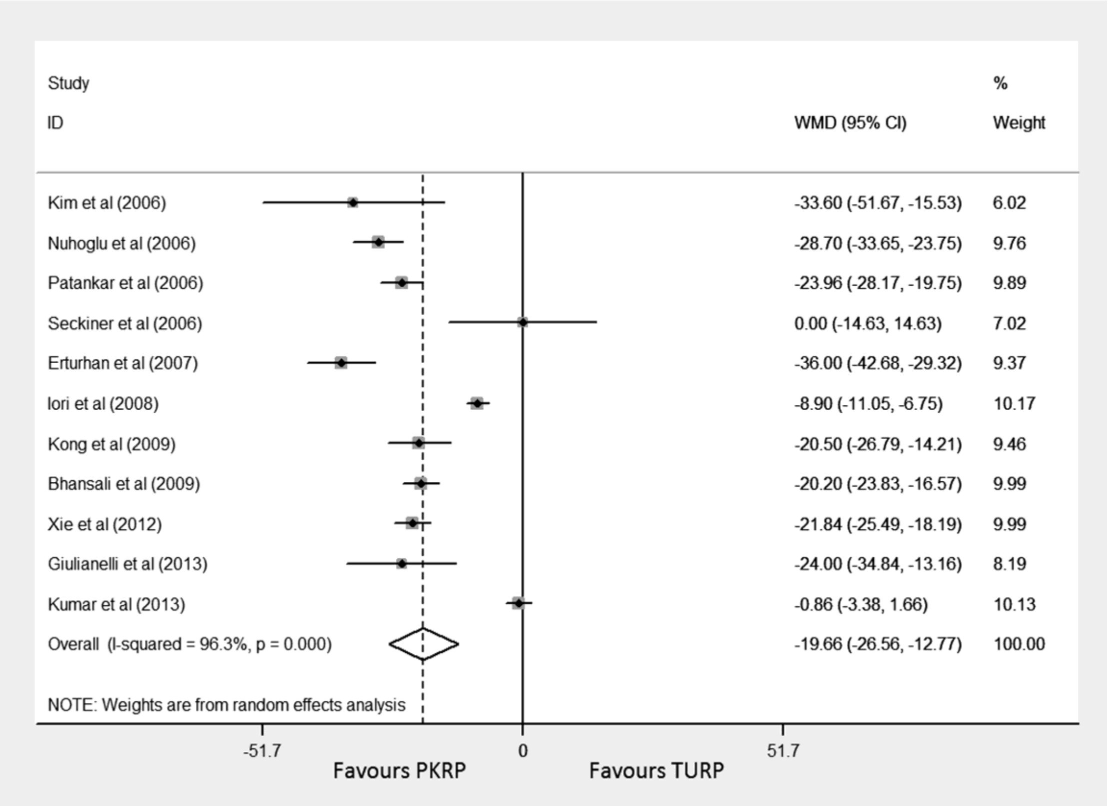

Fig S10 Forest plot of time catheter removal (hours)

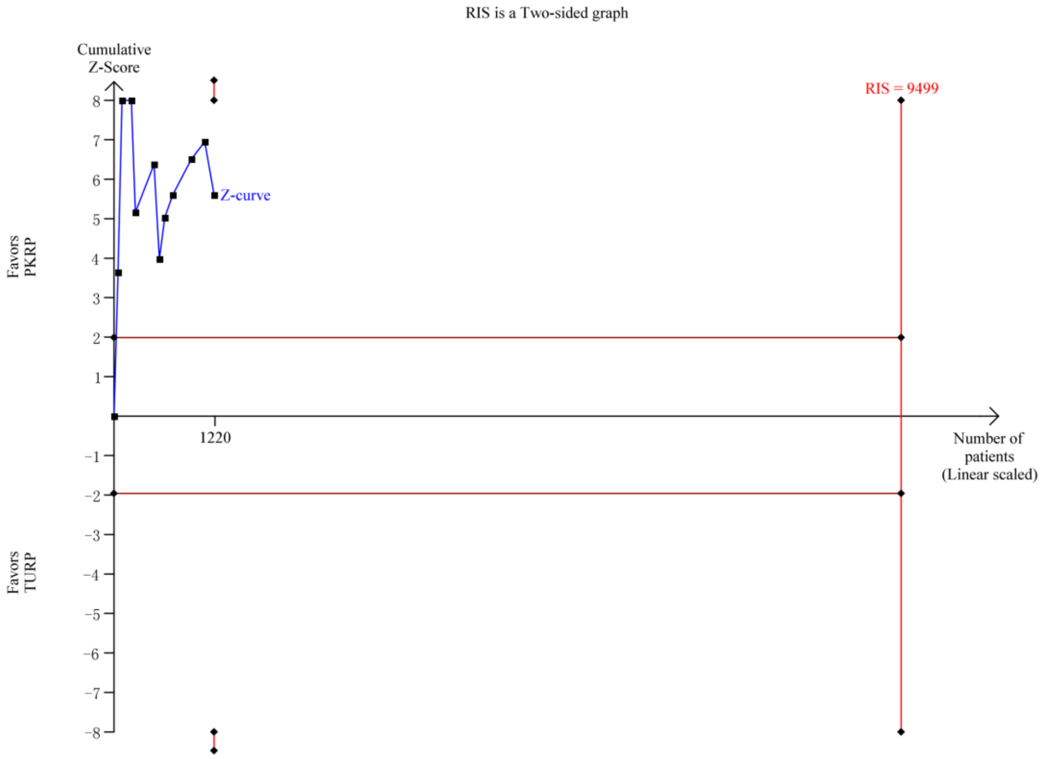

Fig S11 Trial sequential analysis of time catheter removal

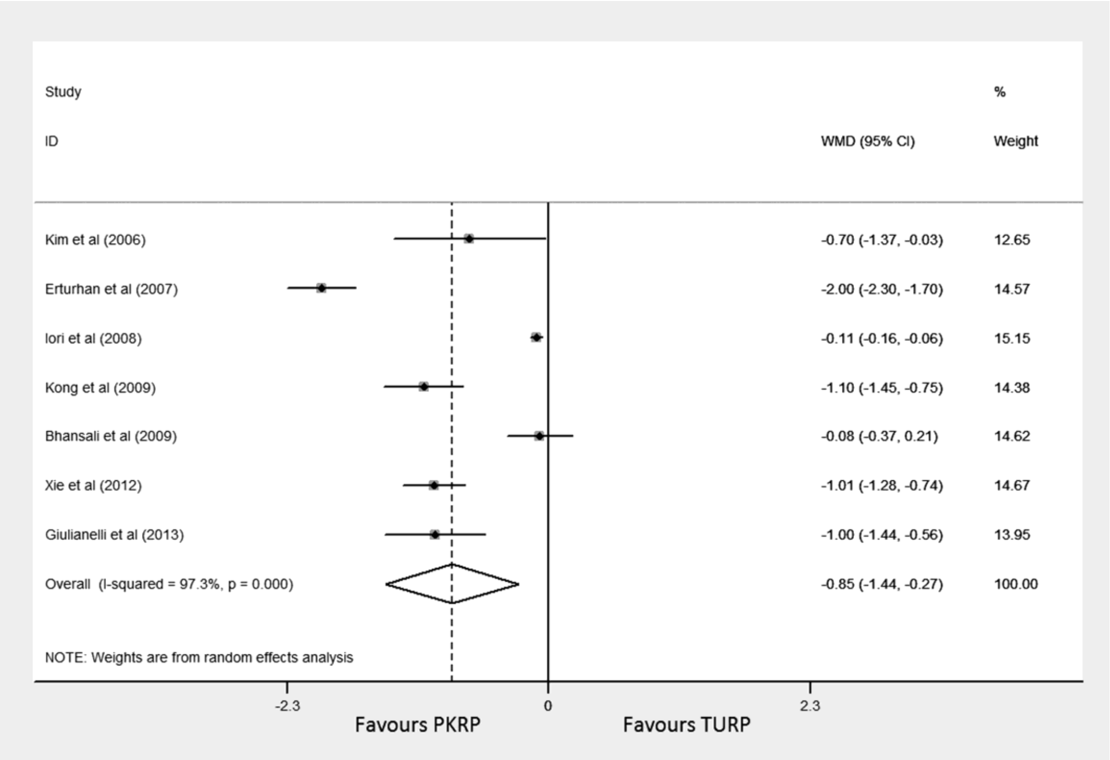

Fig S12 Forest plot of Hospital stay (days)

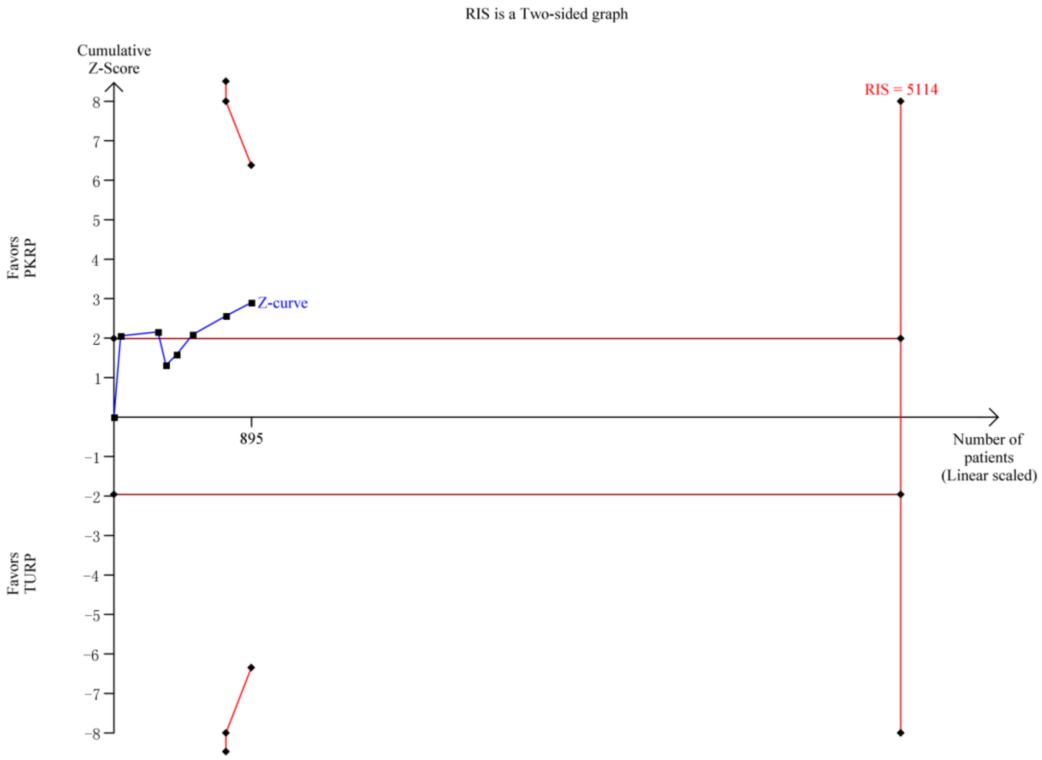

Fig S13 Trial sequential analysis of hospital stay

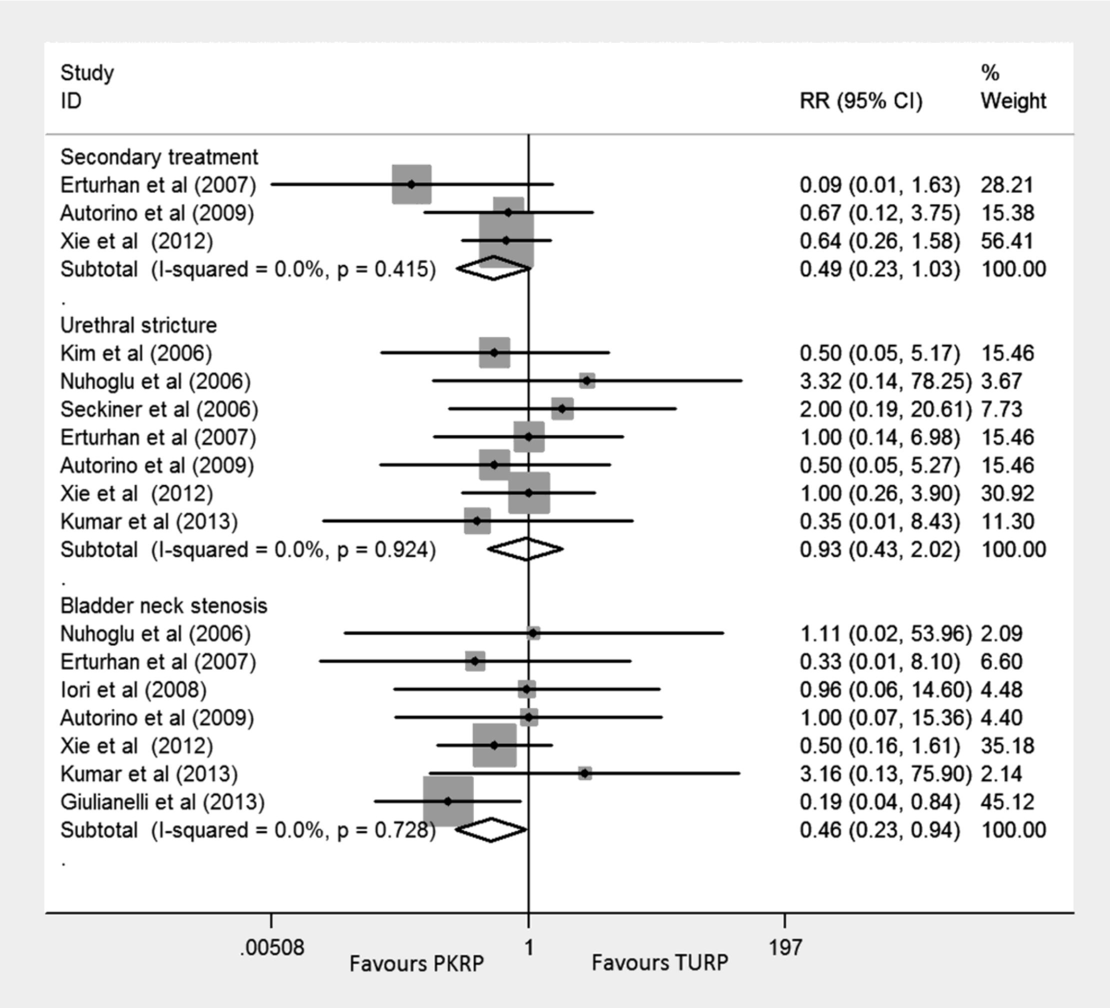

Fig S14 Forest plots of long-term postoperative complications
